# Short telomere length is associated with renal impairment in Japanese subjects with cardiovascular risk

**DOI:** 10.1371/journal.pone.0176138

**Published:** 2017-04-25

**Authors:** Kazuo Eguchi, Lawrence S. Honig, Joseph H. Lee, Satoshi Hoshide, Kazuomi Kario

**Affiliations:** 1 Division of Cardiovascular Medicine, Department of Medicine, Jichi Medical University, Tochigi, Japan; 2 Department of Neurology, Columbia University College of Physicians & Surgeons, New York, NY, United States of America; 3 Sergievsky Center/Taub Institute, Columbia University, New York, NY, United States of America; Shanghai Institute of Hypertension, CHINA

## Abstract

**Introduction:**

Short telomere length has been suggested to be associated with atherosclerotic changes in Western populations. We examined the relationships between leukocyte telomere length and cardiovascular and renal function in a Japanese cohort.

**Participants and methods:**

We enrolled 770 subjects who each had at least one cardiovascular risk factor. The mean age was 59.5 ± 12.2 years; mean BMI was 25.1 ± 4.6 kg/m^2^. We measured leukocyte telomere length (LTL) by quantitative PCR (T/S ratio), and measured other biomarkers from blood and urine samples. In addition, we assessed surrogate markers of arterial stiffness, cardiovascular organ damage and kidney function, including flow-mediated vasodilation (FMD), pulse wave velocity (PWV), carotid artery augmentation index (CAAI), and urinary albumin creatinine ratio (UACR) and eGFR.

**Results:**

Leukocyte telomere length (T/S ratio) was inversely associated with age (r = -0.194, P<0.001), and was lower in men (1.13 ± 0.29%) than in women (1.20 ± 0.31%, P = 0.002). T/S ratio was positively associated with BMI in women (r = 0.11, P = 0.047), but not in men. LTL did not show a significant relationship to cardiovascular surrogate markers, including arterial stiffness, FMD, and PWV, but did show some relationship to CAAI, which was inversely associated with T/S ratio only in men (r = -0.159, P = 0.015). LTL did show a significant positive association with renal function measured by eGFR (r = 0.16, P<0.001) both in men and women.

**Conclusions:**

In this Japanese sample of persons with increased cardiovascular risk, telomere length showed a relationship of longer telomere length to better renal function, but did not overall show convincing association with cardiovascular measures of arterial stiffness and target organ damage.

## Introduction

Telomeres, repetitive TTAGGG sequences at the end of chromosome, protect the integrity of the chromosome. Telomere length shortens with repeated cell divisions [1]. When telomeres become too short, cells may be unable proliferate, and shortened telomeres have been connected with aging and various disease conditions including cardiovascular disease, dementia, aplastic anemia, and pulmonary fibrosis [2].

There have been numerous publications that leukocyte telomere length (LTL) shortening was associated with cardiovascular (CV) diseases and CV risk factors mostly in Western populations [3–8]. Some CV risk factors such as hypertension are associated with short LTL [5], and multiple CV risk factors have been found to be associated with short LTL [7]. In a longitudinal study of coronary artery disease, risk factors associated with LTL shortening were long baseline LTL, age, male sex, and abdominal obesity [9]. LTL shortening has been suggested to be a comprehensive marker of vascular aging [10, 11]. In Asian populations, associations have been found between short LTL and worse prognosis of hypertension [12] and increased risk of stroke [13]. However, overall the clinical significance of LTL as a cardiovascular indicator has not been entirely consistent [14], and it has not been established as a robust cardiovascular risk marker in clinical practice. In the present study, we examined Japanese subjects with at least one known cardiovascular risk factor to determine the relationship between LTL and cardiovascular target organ damage and cardiovascular risk factors in this population which has one of the highest life expectancies in the world.

## Materials and methods

All of the subjects in this study were participants enrolled from the cardiology clinics in the Jichi Medical University, located in Tochigi prefecture, Japan, and the International University of Health and Welfare Hospital from March 2011 to August 2013. This is a cross-sectional study of a clinic-based, observational cohort study, which is also set to be prospectively followed. The institutional review board of Jichi Medical University (#A09-75) and International University of Health and Welfare Hospital (#FK-31) approved this study, and written informed consent was obtained from all participants.

### Study population

Study subjects had at least one of the following cardiovascular risk factors: hypertension, impaired glucose tolerance or diabetes, dyslipidemia, smokers (including those with chronic obstructive pulmonary disease), chronic renal disease, atrial fibrillation, metabolic syndrome (MetS), or sleep apnea syndrome, and histories of cardio- and/or cerebrovascular diseases were enrolled. Subjects with current hemodialysis treatment, chronic inflammatory disease, and malignancy were excluded in this study.

### Definitions

Hypertension was defined as clinic systolic BP (SBP) ≥140 mmHg and/or diastolic BP (DBP) ≥90 mmHg, or the patient being on antihypertensive medication. Impaired fasting glucose was defined as fasting glucose levels ≥110 mg/dL, and impaired glucose tolerance was defined as glucose levels of ≥140 mg/dL at 2 h after a 75-g oral glucose tolerance test (OGTT). In the present study, diabetes was defined as one or more of the following: self-report, the use of diabetes medication, fasting plasma glucose (FPG) ≥126 mg/dL, or hemoglobin A1c (HbA1c) (NGSP) ≥6.5%. The diagnosis of type 2 diabetes was based on the current American Diabetes Association’s criteria [15, 16]. Dyslipidemia was defined as one or more of the following: self-report, total cholesterol level ≥ 240 mg/dL, triglycerides (TG) ≥150 mg/dL, high-density lipoprotein (HDL) <40 mg/dL, or a treatment for hyperlipidemia [17]. MetS was defined according to the guidelines of the Examination Committee of Criteria for Metabolic Syndrome in Japan [18, 19]. Sleep apnea syndrome was defined as an apnea-hypopnea index of ≥15 events/hour as measured by overnight sleep polysomnography [20].

## Measurements

### Clinic BP measurement

Clinic BP was measured at the clinics using a single, consistent device (HEM-5001; Omron Healthcare, Kyoto, Japan), and was performed using three consecutive measurements. The second and the third measurement of BP readings were averaged and used for the analyses.

### Measures of target organ damage

Blood and urine samples were collected in the morning in a fasting state at the baseline and at the 6^th^ month after. The blood samples were centrifuged at 3,000 g for 15 min at room temperature. Plasma/serum samples after separation and urine samples were stored at 4°C in refrigerated containers and sent to a commercial laboratory (SRL Inc., Tokyo, Japan) within 24 hrs. All assays were performed within the 24 hours and was performed at a single laboratory. The urinary albumin level was measured using a turbidimetric immunoassay (SRL Inc.), and expressed as the urinary albumin excretion ratio (UACR, mg/g·Cr). Both serum and urine creatinine were measured by enzymatic assay [21]. The estimated glomerular filtration rate (eGFR) was calculated using a validated equation based on the modified version of the Modification of Diet in Renal Disease (MDRD) study: eGFR (ml/min/1.73m^2^) = 194 x Age ^-0.287^ x S-Cr ^-1.094^ (if female x 0.739) [21]. Chronic kidney disease (CKD) was defined as eGFR<60ml/min/1.73m^2^.

Arterial stiffness was assessed by brachial–ankle pulse wave velocity (baPWV), and arterial wave reflection was assessed by radial augmentation index (AI). The baPWV was measured using a volume-plethysmographic device with four cuffs fitted with oscillometric sensors (form/BP-203RPE II; Omron Healthcare, Kyoto, Japan). The radial AI (rAI) was measured with a semi-automatic tonometry device (Omron 9000AI; Omron Healthcare, Kyoto, Japan) that was wrapped around the wrist to record radial waveforms, which were then calibrated against the contralateral brachial BP measured by an arm cuff immediately after tonometry. This method employs an algorithm based on a linear regression model to estimate central SBP (cSBP) from the “late systolic shoulder” (pSBP2) of the radial pulse waveform, which has been shown to agree closely with cSBP [22]. The Omron 9000AI was used to calculate the peripheral augmentation index as (P2-DBP)/(P1-DBP), taking P1 and P2 as the first and second inflection points on the radial pulse waveform [22]. Methodologic issues related to the reproducibility of baPWV [23], the validity of baPWV [24] and tonometric measurement of carotid–femoral PWV [25] were reported previously.

### Flow-Mediated Dilatation (FMD)

FMD was measured with the standard technique according to the guidelines for ultrasound assessment of the FMD of the brachial artery [26]. Using a 10-MHz linear array transducer probe, the longitudinal image of the right brachial artery was recorded at baseline and then continuously from 30 s before to at least 2 min after the cuff deflation that followed suprasystolic compression (50 mmHg above systolic blood pressure [SBP]) of the right forearm for 5 min. The diastolic diameter of the brachial artery was determined semi-automatically using an instrument equipped with software for monitoring the brachial artery diameter (UNEX Co., Ltd., Nagoya, Japan).

FMD was estimated as the percent change in the diameter over the baseline value at maximal dilatation during reactive hyperemia. Detailed descriptions of this method using the same system have been reported previously [27, 28]. The reproducibility study of FMD measurement (on visits 1and 2) was performed in 32 subjects who did not change the medication between the visits. Pearson’s correlation coefficient of the FMD between visits 1 and 2 was 0.91, p<0.001, and the coefficient of variation was 11%.

### Telomere length

DNA was isolated by using the Puregene DNA purification system (Gentra) from whole blood of venous blood [29]. Telomere length of coded DNA samples, were measured by laboratory personnel blinded to any information other than a coded identifier. A single-plate quantitative PCR method based on the methods of Cawthon et al [30]. This method, previously described by Honig et al [31] was used. In brief, both telomere (T) and single copy gene (S) amplifications in triplicate were performed on the same 384-well plate, with intraplate reference standard DNA samples and “calibrator samples” for correction of inter-plate variability. Forward and reverse primers for telomere sequence were: 5’- CGGTTTGTTTGGGTT TTGGGTTTGGGTT-3’ and 5’-GGCTTGCCTTACCCTTACCCTTACCCTTACCCTTACCCT-3’, and for single copy gene (beta-globin) were 5’- GCTTCTGACACAACTGTGTTCACTAGC-3’ and 5’- CACCAACTTCATCCACGTTCACC-3’. Thermocycling parameters included 95°C x 10min activation, followed by 34 cycles of 95°C x 15 sec and 55°C x 120 sec. Telomere to single copy (T/S) ratio was calculated by average of the triplicate samples; coefficient of variance was 5 to 8%.

## Statistical analysis

All statistical analyses were carried out with IBM SPSS Statistics software, version 19 (IBM Inc., Armonk, NY). As the UACR distributions were highly positively skewed, these parameters were log-transformed before statistical analyses. A two-tailed paired *t*-test was used to compare the mean values before and after each drug therapy. Data are expressed as the mean ° SD or a percentage. Pearson’s correlation coefficients (“r”) were used to calculate the correlation between the changes in BP parameters and the changes in the measures of target organ damage. Multiple linear regression analyses were performed for creatinine and eGFR as dependent variables using the following selected independent variables: age, sex (0 = women, 1 = men), body mass index, LTL (TS ratio, %), current and past smoking (yes = 1, no = 0), diabetes mellitus (yes = 1, no = 0), dyslipidemia (yes = 1, no = 0), and the presence of hypertension (yes = 1, no = 0). Values of *p*<0.05 were considered to indicate statistical significance. In this exploratory analysis, there were no corrections for multiple comparisons.

## Results

The characteristics of subjects are shown in **Table 1**. The mean age was 59.5 ± 12.2 years, and the telomere length, measured as the adjusted telomere-to-single copy gene (T/S) ratio (%)) was significantly longer in females than in males (1.20 ± 0.31 vs. 1.13 ± 0.29, respectively; P = 0.002).

**Table 1:** Patient characteristics.

	Female (n = 321)	Male (n = 445)	p-Value
**Age (yrs)**	59.7 ± 12.8	59.4 ± 11.8	0.72
**Body mass index (kg/m**^**2**^**)**	25.1 ± 5.8	25.1 ± 3.4	0.91
**Current smoking (%)**	6.2	25.2	<0.001
**Current or past smoking (%)**	16.2	56.4	<0.001
**Habitual drinking (%)**	12.1	55.2	<0.001
**History of coronary heart disease (%)**	11.5	24.7	<0.001
**Duration of hypertension (yrs)**	6.9 ± 8.2	6.4 ± 7.3	0.38
**Hypertension (%)**	91.9	91	0.70
**Diabetes mellitus (%)**	24.3	27.3	0.62
**Dyslipidemia (%)**	46.1	51.9	0.12
**High uric acid (%)**	11.8	39.3	<0.001
**eGFR (ml/min/1.73m**^**2**^**)**	79.3 ± 23.5	72.8 ± 19	<0.001
**Chronic kidney disease (eGFR<60) (%)**	17.1	21.8	0.12

Habitual drinking was defined as drinking more than 5 days a week. High uric acid was defined as uric acid level ≧7.0 mg/dL. eGFR, estimated glomerular filtration rate.

**Fig 1:**
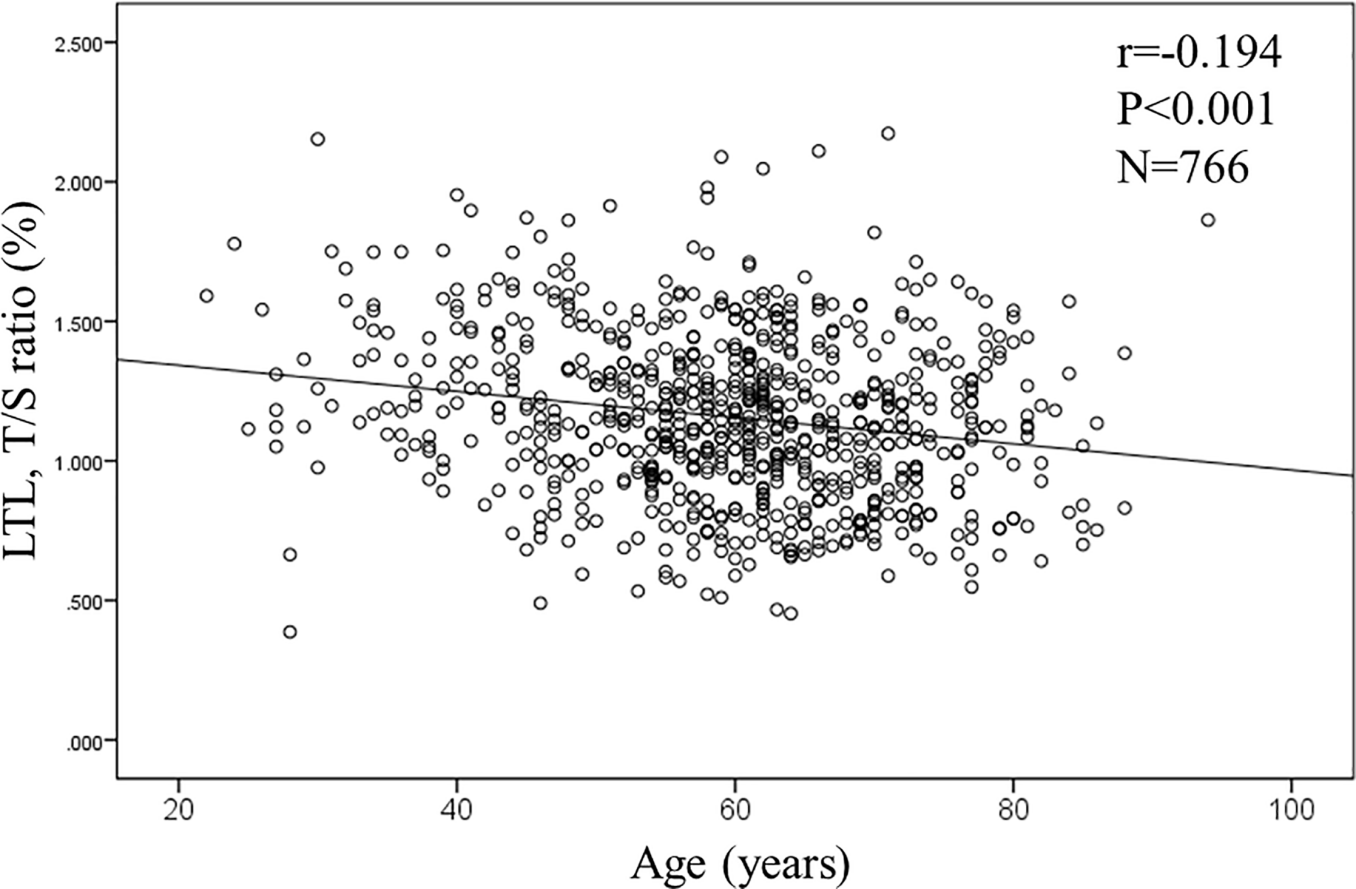
Correlation between age and leukocyte telomere length (LTL).

**Fig 2:**
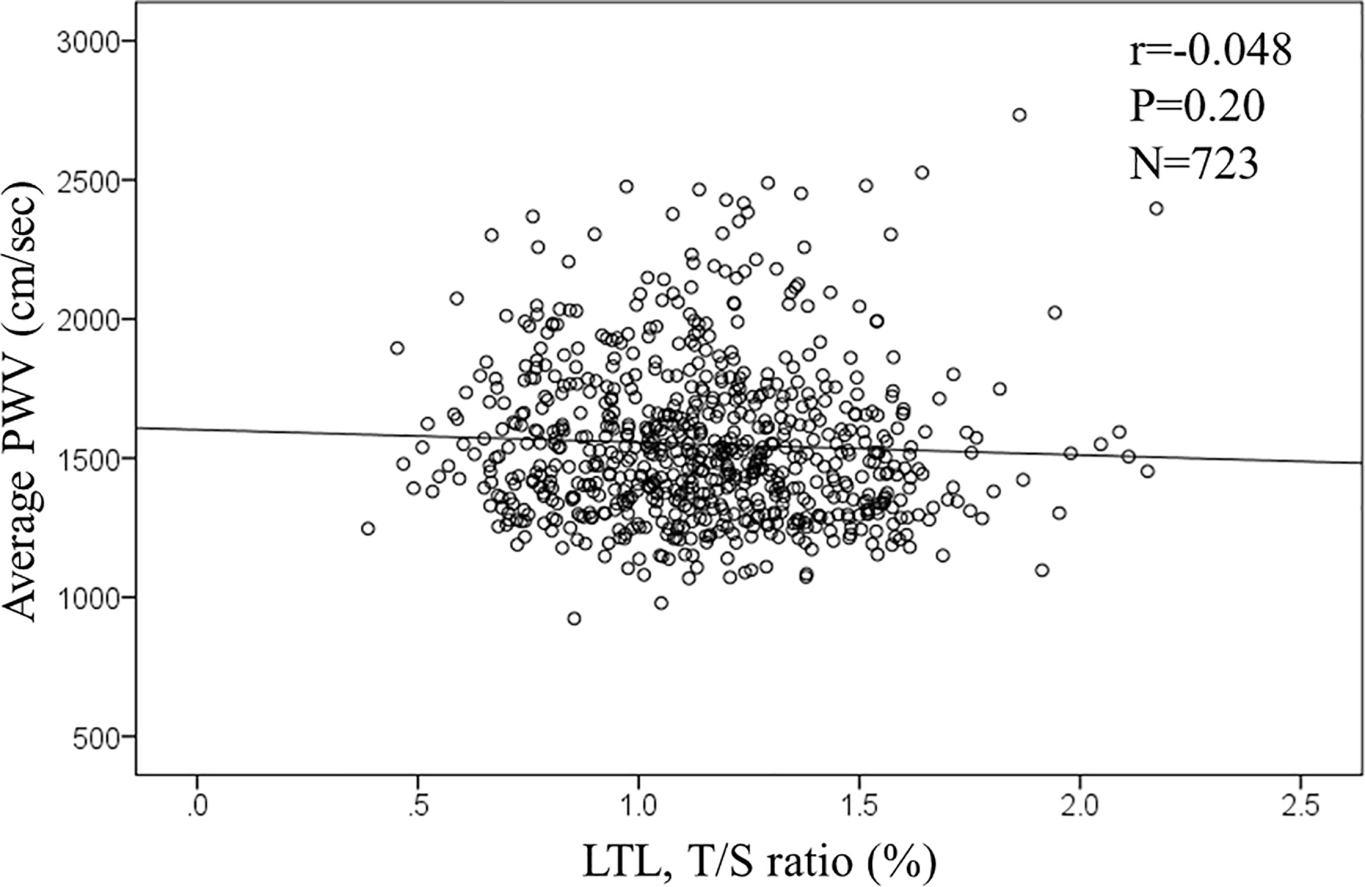
Correlation between leukocyte telomere length (LTL) and Pulse wave velocity.

**Fig 3:**
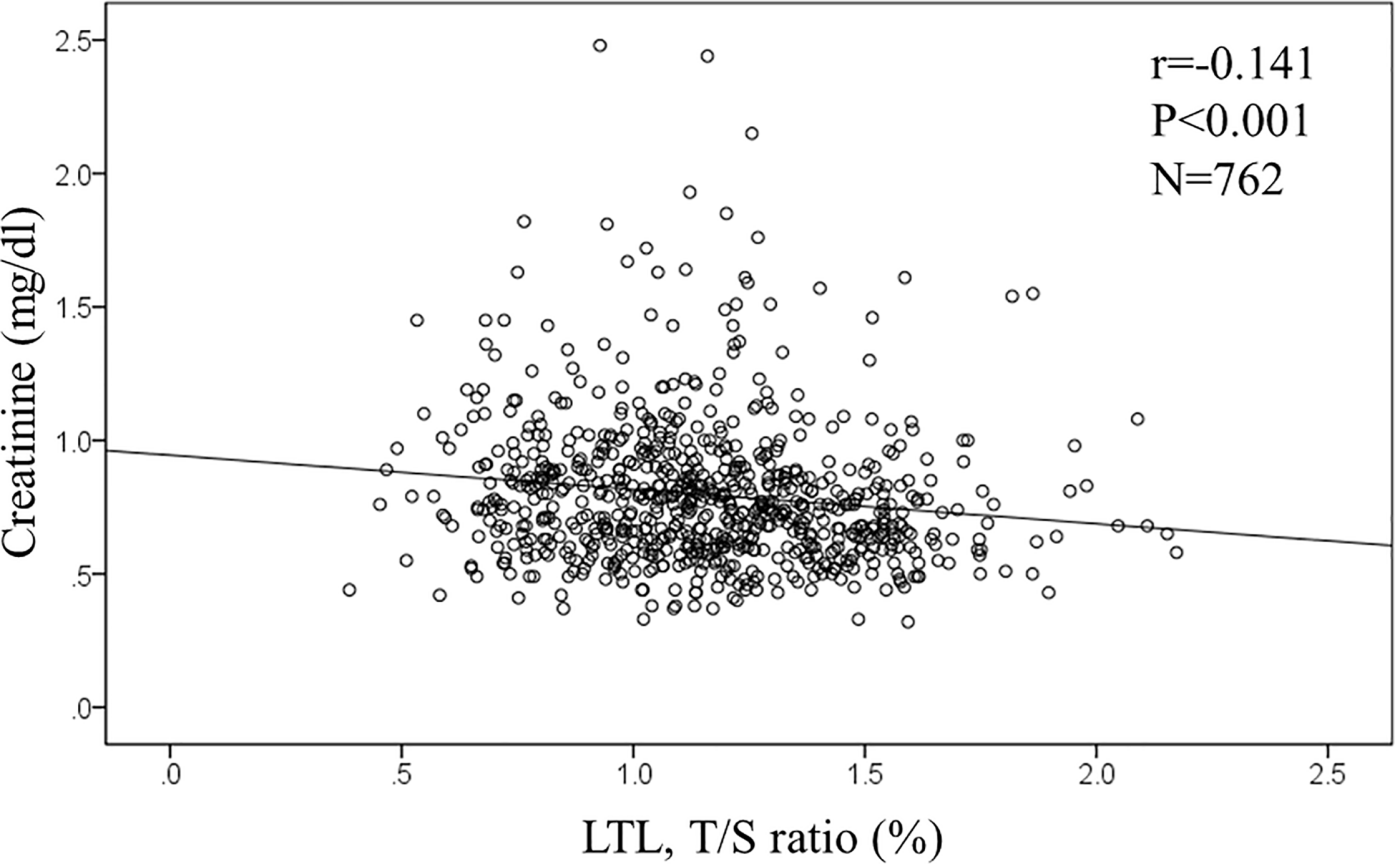
Correlation between leukocyte telomere length (LTL) and serum creatinine.

In the overall population, age and the level of serum creatinine were significantly associated with telomere length. The T/S ratio was negatively associated with both age (r = -0.194, p<0.001, n = 766; **Fig 1**) and levels of serum creatinine (r = -0.141, p<0.001, n = 762; **Fig 2**). However, levels of PWV was not associated with the T/S ratio (r = -0.048, p = 0.20, n = 723; **Fig 3**). Similarly, measures of rAI (r = 0.038, p = 0.308, n = 729) and FMD (r = 0.02, p = 0.967, n = 687) were not associated with the T/S ratio.

When analyzed separately by gender (**Table 2**), some traits were significantly associated with the T/S ration in both genders, but we identified additional traits that were associated with the T/S ratio in only one gender.

**Table 2:** Correlations between adjusted T/S ratio and demographic or clinical traits.

	Female (n = 321)		Male (n = 445)	
	r	p-Value	r	p-Value
**Age (years)**	-0.20	<0.001	-0.19	<0.001
**Body mass index (kg/m**^**2**^**)**	0.11	0.047	-0.01	0.809
**Current smoking (Yes = 1, No = 0)**	-0.02	0.70	0.01	0.81
**Drinking alcohol (Yes = 1, No = 0)**	0.07	0.19	-0.01	0.775
**History of hypertension (years)**	-0.07	0.18	0.01	0.87
**Diabetes (Yes = 1, No = 0)**	0.00	0.97	0.03	0.52
**Dyslipidemia (Yes = 1, No = 0)**	-0.04	0.50	0.04	0.41
**Chronic kidney disease (Yes = 1, No = 0)**	-0.1	0.062	-0.01	0.86
**Coronary artery disease (Yes = 1, No = 0)**	-0.03	0.60	-0.04	0.44
**HbA1c (%)**	-0.02	0.66	-0.09	0.06
**LDL cholesterol (mg/dl)**	-0.05	0.38	-0.05	0.33
**Uric acid (mg/dl)**	-0.01	0.88	0.00	1.00
**Serum creatinine (mg/dl)**	-0.15	0.009	-0.07	0.13
**Estimated GFR (ml/min)**	0.13	0.019	0.15	0.001
**Urinary albumin creatinine ratio**^**1)**^ **(mg/g·cr)**	-0.03	0.61	-0.03	0.49
**Flow mediated dilatation (%)**	0.08	0.19	-0.08	0.12
**Brachial SBP (mmHg)**	0.05	0.36	0.04	0.38
**Brachial DBP (mmHg)**	0.12	0.044	0.07	0.13
**Brachial PP (mmHg)**	-0.09	0.11	-0.02	0.68
**Pulse rate (bpm)**	-0.06	0.28	0.03	0.58
**Radial AI (%)**^**2)**^	0.00	0.93	0.03	0.53
**Radial AI adjusted by HR 75 (%)**^**2)**^	-0.03	0.58	0.03	0.50
**Central SBP (mmHg)**^**2)**^	0.07	0.20	0.05	0.29
**baPWV (cm/sec)**^**3)**^	-0.11	0.067	0.00	0.97
**Carotid AI (%)** ^**4)**^	0.04	0.55	-0.16	0.02

AI, augmentation index, PWV, pulse wave velocity. 1) n = 311 in females and 439 in males; 2) n = 302 in females and 421 in males; 3) n = 302 in females and 421 in males;4) n = 195 in females and 234 in males.

### Cardiovascular risk factors

As expected, age was associated with the T/S ratio in both genders. BMI was associated with longer telomere length in females only (r = 0.11, p = 0.047, n = 321). However, for the remaining potential risk factors—such as smoking, drinking, HbA1c, LDL cholesterol and uric acid, and the history of hypertension, diabetes, or kidney disease–no significant association was observed with the T/S ratio. However, for the indicators of target organ damage, we observed that the levels of serum creatinine (r = -0.15, p = 0.0009) were associated with the T/S ratio in females, but not in males (r = -0.07, p = 0.13). The relationship in females remained significant when adjusted by age, BMI, current and past smoking, diabetes, dyslipidemia, and hypertension (S1 Table). The levels of estimated GFR were associated with the T/S ratio in both females (r = 0.13, p = 0.019) and in males (r = 0.15, p = 0.001), and these results remained significant even when adjusted by BMI, current and past smoking, diabetes, dyslipidemia, and hypertension (S2 Table). Among hypertension measures, only brachial DBP was significantly associated with the T/S ratio in females (r = 0.12, p = 0.044), but not in males.

We ran additional analyses by the presence of CKD (eGFR above or below 60 ml/min/1.73m^2^). The correlation coefficients between LTL and serum creatinine were significant in the non-CKD group (r = - 0.123, p = 0.002 n = 611), but not significant in the CKD group (r = 0.067, p = 0.413, n = 151). Similarly, the correlation coefficients between LTL and eGFR were significant in the non-CKD group (r = 0.098, p = 0.015, n = 611), but not in the CKD group (r = - 0.032, p = 0.699, n = 151).

When each medication used at the time of LTL measurement (S3 Table) was further adjusted, the significant relationship between LTL and serum creatinine, and LTL and eGFR remained significant (S4 Table).

### Cardiovascular measurements

Carotid AI, a measure of atrial stiffness, was negatively associated with the T/S ratio in males only (r = -0.16, p = 0.015, n = 234). Other measures to assess FMD were not associated with the T/S in either genders (female: r = 0.08, p = 0.19, n = 286; male: r = -0.08, p = 0.12, n = 401). Similarly, the relationships between the T/S ratio and PWV were not found to be significant in females (r = -0.11, p = 0.067, n = 302) and in males (r = -0.002, p = 0.97, n = 421).

## Discussion

LTL in Japanese subjects with cardiovascular risk factors was associated with age and gender, as is usually found, but also associated, at least in a simple model, with markers of renal aging (serum creatinine and eGFR). Overall, despite some single-sex borderline associations LTL was not associated with the cardiovascular measures of endothelial function and vascular stiffness. This study is one of the largest studies that examining the relationship between LTL and measures of cardiovascular damage in a Japanese population sample.

### LTL and PWV

In the present study, LTL was not associated with baPWV in overall population. However, T/S ratio showed a trend towards association with PWV in females (r = -0.11, p = 0.067, n = 302), but not in males. This is in contrast to the observation that LTL was associated with aortic PWV in men, but not in women [32]. In a study of persons of African descent, significant relationship between LTL and aortic stiffness was observed in both genders, but was also seen in premenopausal women.[33]. It is possible that telomere length in white blood cells may change in parallel with that of vascular cells [34]. Also, a major pathophysiological contributor to atherosclerosis is chronic inflammation [35], which could accelerate the turnover of white blood cells leading to telomere attrition. The reasons why we have not found significant relationship between cardiovascular factors and LTL in the present study, while other studies have, are not clear, but it could be speculated that the Japanese population may have less atherosclerosis-associated inflammation than Western populations [36].

### LTL and central aortic hemodynamics

In the present study, LTL was not associated with central aortic parameters except for a weak correlation with carotid AI in males. This is in agreement with the results of recent publication by Raymond and colleagues [33]. Central hemodynamic parameters—specifically rAI, central BP, and PP—could change by various conditions such as treatment, pulse rate, or vascular stiffness at the time of measurements. Furthermore, rAI could be paradoxically decreased in diabetes or obesity [37, 38]. Therefore, central hemodynamics may not be a very robust measure of vascular aging when it is analyzed as cross-sectional study.

### LTL and FMD

In the present study, LTL was not associated with FMD in both genders. This is in agreement with the results of Nakashima et al [34]. FMD could also be affected by various factors such as medication, physical activity, or foods. Nezu et al [39], also found that telomere length was not associated with FMD, but found that a different telomere measurement (G-tail length) was significantly associated with FMD. In the present study, FMD was measured in fasting and non-medication conditions by one trained technician. Even though the subjects in our study is heterogeneous and mostly on treatment, LTL was not a good surrogate of vascular function in our study.

### LTL and renal function

In the overall population sample, LTL was positively associated with serum creatinine level. When it was analyzed by genders, LTL was significantly associated with serum creatinine in females, but not in males. LTL was also associated with eGFR in both sexes, independently of other covariates (S2 Table). Because eGFR indicates renal function more accurately, we consider that LTL is a marker of renal aging in both sexes independently of age and other risk factors. With regard to creatinine, LTL was associated with creatinine in females, but not in males. However, the correlation showed the same trend even in males and in overall subjects, and LTL was independently associated with creatinine when it was adjusted by gender. The significant relationships between LTL and serum creatinine, and LTL and eGFR were independent of each medication used in the subjects. Therefore, the LTL is basically associated with renal function. On the other hand, LTL was not associated with UACR.

Association between LTL and renal function has been reported in several studies[40–42]. Shorter telomere length was associated with renal dysfunction evaluated by eGFR in chronic heart failure patients [40]. LTL was associated with baseline eGFR evaluated by creatinine in patients with coronary heart disease [41]. In longitudinal studies, LTL has been found to be a marker of CKD progression [42]. And lower baseline GFR has been reported to be a marker of more rapid telomere shortening [41], but the relationship was diminished by adjusting for age. It has been suggested that the kidney may be a particularly sensitive marker of biological age [43], and the present study might support the notion that aging of the kidney is a good marker for biological aging. The insignificant relationship between LTL and renal function in subjects with CKD (but significant in those with non-CKD) may indicate that LTL and renal functions are markers of biological aging in the early stage of biological aging.

There are some limitations in this study. Correlations between the telomere length and parameters used in this study were weak, but the reason for the weak correlation between the telomere length and parameters used in this study are unclear. We speculate that inter-individual differences of telomere length may be large, and telomere length was measured by leukocytes but not directory from kidney cells or arteries. References #40 and #41 studied the relationship between LTL and kidney function, and the extent of correlation was not very large. In ref #40, Pearson’s correlation coefficient for the association between LTL and eGFR was 0.123 (p<0.001), and in ref #41, there were no association between LTL and serum creatinine, Ccr, eGFR, etc in cross-sectional analyses.

In conclusion, in this study in Japanese subjects with cardiovascular risk factors, leukocyte telomere length was not associated with measures of arterial stiffness and cardiovascular target organ damage, but was associated with renal function. While additional analyses are in progress, it is possible that in the Japanese population, telomere length may be a marker of renal aging more so than cardiovascular target organ damage.

## Supporting information

S1 Table Factors associated with serum creatinine. Multiple linear regression analyses were performed for serum creatinine as a dependent variable. LTL, leukocyte telomere length.
(DOCX)

S2 Table Factors associated with estimated GFR. Multiple linear regression analyses were performed for eGFR as a dependent variable. Because age was used for calculating eGFR, age was not used as an independent variable. LTL, leukocyte telomere length.
(DOCX)

S3 Table Medications used at the LTL measurement. (DOCX)

S4 Table Factors associated with serum creatinine and eGFR. Each model was adjusted by age, BMI, current and past smoking, diabetes, dyslipidemia, hypertension, and each medication. LTL, leukocyte telomere length, TS, telomere to single copy.
(DOCX)
