# Supplementary material for: Short telomere length is associated with renal impairment in Japanese subjects with cardiovascular risk
Source: PLoS One. 2017 Apr 25;12(4):e0176138. doi: 10.1371/journal.pone.0176138 (PMC5404870; doi:10.1371/journal.pone.0176138)
Supplement: S1 Table — Multiple linear regression analyses were performed for serum creatinine as a dependent variable. LTL, leukocyte telomere length. (DOCX) [file pone.0176138.s001.docx]

| **S1 Table. Factors associated with serum creatinine** | | |  |  |  |  |
| --- | --- | --- | --- | --- | --- | --- |
|  | **Overall** | | **Female** | | **Male** | |
|  | **Beta** | **p-value** | **Beta** | **p-value** | **Beta** | **p-value** |
| **Sex (male=1, female=0)** | 0.45 | <0.001 |  |  |  |  |
| **Age (yrs)** | 0.10 | 0.002 | 0.11 | 0.075 | 0.15 | 0.003 |
| **Body mass index (kg/m^2^)** | 0.07 | 0.03 | -0.03 | 0.62 | 0.20 | <0.001 |
| **LTL (TS ratio, %)** | -0.08 | 0.02 | -0.12 | 0.031 | -0.04 | 0.35 |
| **Current and past smoking (yes=1, no=0)** | -0.01 | 0.69 | 0.05 | 0.36 | -0.05 | 0.26 |
| **Diabetes mellitus (yes=1, no=0)** | 0.05 | 0.13 | 0.12 | 0.038 | 0.06 | 0.20 |
| **Dyslipidemia (yes=1, no=0)** | 0.09 | 0.01 | 0.04 | 0.46 | 0.08 | 0.085 |
| **Hypertension (yes=1, no=0)** | 0.04 | 0.24 | 0.04 | 0.53 | 0.04 | 0.43 |
| Multiple linear regression analyses were performed for serum creatinine as a dependent variable. | | | | | |  |
| LTL, leukocyte telomere length |  |  |  |  |  |  |
