# Supplementary material for: Short telomere length is associated with renal impairment in Japanese subjects with cardiovascular risk
Source: PLoS One. 2017 Apr 25;12(4):e0176138. doi: 10.1371/journal.pone.0176138 (PMC5404870; doi:10.1371/journal.pone.0176138)
Supplement: S2 Table — Multiple linear regression analyses were performed for eGFR as a dependent variable. Because age was used for calculating eGFR, age was not used as an independent variable. LTL, leukocyte telomere length. (DOCX) [file pone.0176138.s002.docx]

| **S2 Table. Factors associated with estimated GFR** | |  |  |  |  |  |
| --- | --- | --- | --- | --- | --- | --- |
|  | **Overall** | | **Female** | | **Male** | |
|  | **Beta** | **p-value** | **Beta** | **p-value** | **Beta** | **p-value** |
| **Sex (male=1, female=0)** | -0.14 | <0.001 |  |  |  |  |
| **Body mass index (kg/m^2^)** | 0.03 | 0.47 | 0.12 | 0.03 | -0.11 | 0.02 |
| **LTL (TS ratio, %)** | 0.15 | <0.001 | 0.12 | 0.03 | 0.16 | 0.001 |
| **Current and past smoking (yes=1, no=0)** | 0.03 | 0.51 | -0.003 | 0.96 | 0.04 | 0.41 |
| **Diabetes mellitus (yes=1, no=0)** | -0.07 | 0.065 | -0.11 | 0.07 | -0.07 | 0.12 |
| **Dyslipidemia (yes=1, no=0)** | -0.15 | <0.001 | -0.13 | 0.03 | -0.13 | 0.006 |
| **Hypertension (yes=1, no=0)** | -0.07 | 0.038 | -0.105 | 0.06 | -0.04 | 0.39 |
| Multiple linear regression analyses were performed for eGFR as a dependent variable. | | | | |  |  |
| Because age was used for calculating eGFR, age was not used as an independent variable. | | | | | |  |
| LTL, leukocyte telomere length |  |  |  |  |  |  |
