# Supplementary material for: Short telomere length is associated with renal impairment in Japanese subjects with cardiovascular risk
Source: PLoS One. 2017 Apr 25;12(4):e0176138. doi: 10.1371/journal.pone.0176138 (PMC5404870; doi:10.1371/journal.pone.0176138)
Supplement: S3 Table — (DOCX) [file pone.0176138.s003.docx]

| **S3 Table. Medications used at the LTL measurement** | |
| --- | --- |
| **Calcium channel blockers (%)** | 79.2 |
| **Renin angiotensin system inhibitors (%)** | 74.4 |
| **Thiazide diuretics (%)** | 29.8 |
| **β-blockers (%)** | 25.1 |
| **Aldosterone blockers (%)** | 7.7 |
| **α-blockers (%)** | 6.4 |
| **Loop diuretics (%)** | 5.4 |
| **Nitrates (%)** | 6.1 |
| **Statins (%)** | 32.0 |
| **Fibrates (%)** | 2.0 |
| **Ezetimibe (%)** | 1.4 |
| **Ethyl icosapentate (%)** | 1.0 |
| **α-glucosidase inhibitors (%)** | 6.4 |
| **Metformin (%)** | 5.7 |
| **Sulfonylureas (%)** | 5.1 |
| **Dipeptidyl peptidase-4 inhibitors (%)** | 4.7 |
| **Insulin (%)** | 2.2 |
| **Pioglitazone (%)** | 1.3 |
| **Glinides (%)** | 1.3 |
| **Antihyperuricemics (%)** | 11.9 |
| **Aspirin (%)** | 18.8 |
| **Antiplatelets (%)** | 7.6 |
| **Warfarin (%)** | 3.0 |
