# Supplementary material for: Short telomere length is associated with renal impairment in Japanese subjects with cardiovascular risk
Source: PLoS One. 2017 Apr 25;12(4):e0176138. doi: 10.1371/journal.pone.0176138 (PMC5404870; doi:10.1371/journal.pone.0176138)
Supplement: S4 Table — Each model was adjusted by age, BMI, current and past smoking, diabetes, dyslipidemia, hypertension, and each medication. LTL, leukocyte telomere length, TS, telomere to single copy. (DOCX) [file pone.0176138.s004.docx]

| **S4 Table. Factors associated with serum creatinine and eGFR** | | | | | | |  |  |
| --- | --- | --- | --- | --- | --- | --- | --- | --- |
|  |  | **Creatinine** | | **eGFR** | | **Medications adjusted in each model** | | |
|  |  | **Beta** | **P-value** | **Beta** | **P-value** |  |  |  |
| **Model 1** | LTL (TS ratio, %) | -0.076 | 0.02 | 0.078 | 0.022 | Calcium channel blockers | | |
| **Model 2** | LTL (TS ratio, %) | -0.084 | 0.01 | 0.086 | 0.01 | Renin angiotensin system inhibitors | | |
| **Model 3** | LTL (TS ratio, %) | -0.087 | 0.007 | 0.087 | 0.01 | Thiazide diuretics | | |
| **Model 4** | LTL (TS ratio, %) | -0.078 | 0.016 | 0.079 | 0.02 | β-blockers | | |
| **Model 5** | LTL (TS ratio, %) | -0.078 | 0.016 | 0.08 | 0.019 | Aldosterone blockers | | |
| **Model 6** | LTL (TS ratio, %) | -0.078 | 0.017 | 0.08 | 0.018 | α-blockers | | |
| **Model 7** | LTL (TS ratio, %) | -0.076 | 0.018 | 0.078 | 0.021 | Loop diuretics | | |
| **Model 8** | LTL (TS ratio, %) | -0.077 | 0.019 | 0.078 | 0.021 | Nitrates | | |
| **Model 9** | LTL (TS ratio, %) | -0.076 | 0.02 | 0.077 | 0.023 | Statins | | |
| **Model 10** | LTL (TS ratio, %) | -0.079 | 0.016 | 0.081 | 0.018 | Fibrates | | |
| **Model 11** | LTL (TS ratio, %) | -0.077 | 0.019 | 0.078 | 0.022 | Ezetimibe | | |
| **Model 12** | LTL (TS ratio, %) | -0.075 | 0.021 | 0.077 | 0.024 | Ethyl icosapentate | | |
| **Model 13** | LTL (TS ratio, %) | -0.077 | 0.019 | 0.078 | 0.021 | α-glucosidase inhibitors | | |
| **Model 14** | LTL (TS ratio, %) | -0.079 | 0.016 | 0.08 | 0.019 | Metformin | | |
| **Model 15** | LTL (TS ratio, %) | -0.078 | 0.017 | 0.079 | 0.02 | Sulfonylureas | | |
| **Model 16** | LTL (TS ratio, %) | -0.077 | 0.018 | 0.079 | 0.02 | Dipeptidyl peptidase-4 inhibitors | | |
| **Model 17** | LTL (TS ratio, %) | -0.078 | 0.016 | 0.079 | 0.019 | Insulin | | |
| **Model 18** | LTL (TS ratio, %) | -0.077 | 0.019 | 0.078 | 0.021 | Pioglitazone | | |
| **Model 19** | LTL (TS ratio, %) | -0.075 | 0.022 | 0.076 | 0.025 | Glinides | | |
| **Model 20** | LTL (TS ratio, %) | -0.064 | 0.04 | 0.067 | 0.041 | Antihyperuricemics | | |
| **Model 21** | LTL (TS ratio, %) | -0.074 | 0.023 | 0.077 | 0.024 | Aspirin | | |
| **Model 22** | LTL (TS ratio, %) | -0.075 | 0.021 | 0.078 | 0.022 | Antiplatelets | | |
| **Model 23** | LTL (TS ratio, %) | -0.076 | 0.02 | 0.078 | 0.022 | Warfarin | | |
| Each model was adjusted by age, BMI, current and past smoking, diabetes, dyslipidemia, hypertension, and each medication. | | | | | | | | |
| LTL, leukocyte telomere length, TS, telomere to single copy | | | |  | | |  |  |
